# Advancing Gut Microbiome Research: The Shift from Metagenomics to Multi-Omics and Future Perspectives

**DOI:** 10.4014/jmb.2412.12001

**Published:** 2025-03-26

**Authors:** So-Yeon Yang, Seung Min Han, Ji-Young Lee, Kyoung Su Kim, Jae-Eun Lee, Dong-Woo Lee

**Affiliations:** Department of Biotechnology, Yonsei University, Seoul 03722, Republic of Korea

**Keywords:** Gut microbiome, multi-omics, host-microbe interactions, personalized medicine, microbiomebased therapeutics

## Abstract

The gut microbiome, a dynamic and integral component of human health, has co-evolved with its host, playing essential roles in metabolism, immunity, and disease prevention. Traditional microbiome studies, primarily focused on microbial composition, have provided limited insights into the functional and mechanistic interactions between microbiota and their host. The advent of multi-omics technologies has transformed microbiome research by integrating genomics, transcriptomics, proteomics, and metabolomics, offering a comprehensive, systems-level understanding of microbial ecology and host-microbiome interactions. These advances have propelled innovations in personalized medicine, enabling more precise diagnostics and targeted therapeutic strategies. This review highlights recent breakthroughs in microbiome research, demonstrating how these approaches have elucidated microbial functions and their implications for health and disease. Additionally, it underscores the necessity of standardizing multi-omics methodologies, conducting large-scale cohort studies, and developing novel platforms for mechanistic studies, which are critical steps toward translating microbiome research into clinical applications and advancing precision medicine.

## Introduction

Humans have co-evolved with trillions of microorganisms, forming a complex and adaptive gut microbiome that plays a crucial role in metabolism, nutrition, immunity, and disease prevention [1-4]. Over the past few decades, it has been noted that gut dysbiosis—an imbalance in the microbiome—has been associated with various diseases, including inflammatory bowel disease (IBD) [5], multiple sclerosis [6], obesity [7], diabetes [8], allergies [9], autism [10], and cancer [11]. Moreover, recent studies suggest that gut microbiota composition influences the efficacy of therapeutic interventions, particularly in IBD and cancer treatment [12, 13]. Maintaining a balanced gut microbiome is therefore crucial for overall health, as it directly regulates colonic cell metabolism and systemic physiological processes [14, 15].

Traditional microbiome studies have primarily focused on taxonomic profiling, which has significantly contributed to understanding the overall microbial ecosystem in the gut. While compositional shifts in the microbiome have been linked to chronic diseases [16], substantial inter- and intra-individual variability—shaped by factors such as diet, environment, and lifestyle—complicates the ability to establish definitive microbiome-disease associations [17, 18]. These findings underscore the need to move beyond microbial composition and focus on core functional attributes, such as functional redundancy, which ensures ecosystem stability despite taxonomic differences [1]. Additionally, precise characterization of healthy versus dysbiotic microbiomes is essential for developing accurate diagnostic, prognostic, and therapeutic strategies [16, 19]. However, the mechanisms through which a balanced microbiome is maintained via host-microbiome co-evolution remain poorly understood.

Next-generation microbiome research seeks to address these challenges by emphasizing the functional characteristics of gut microbial communities and their interactions with the host [20]. Advances in sequencing technologies and computational tools have enabled large-scale cohort studies that integrate both taxonomic and functional analyses of the microbiome [21, 22]. Notable initiatives, such as the Metagenomics of the Human Intestinal Tract (MetaHIT) project in Europe (http://www.metahit.eu) [23] and the Human Microbiome Project (HMP) in the United States (http://hmpdacc.org) [19], have established extensive reference databases of human gut microbial genes and genomes. These large-scale studies have facilitated the identification of shared functional traits across individuals, shifting the focus from taxonomy-driven analyses to function-based insights [21, 24, 25]. This paradigm shift has paved the way for the integration of multi-omics technologies.

Multi-omics approaches have revolutionized microbiome research by providing a holistic, system-level perspective that transcends the limitations of traditional metagenomics. The Integrative Human Microbiome Project (iHMP, Phase II) [25], has applied multi-omics strategies to investigate the epidemiology, evolution, and diversity of gut microbial communities. These initiatives extend beyond taxonomic and functional profiling, laying the groundwork for microbiome-based disease prevention, therapeutic interventions, and precision medicine [26]. As a result, gut microbiome research has become a cornerstone of future bio-healthcare innovation [27].

Despite these advancements, several challenges remain. Understanding microbial functions within specific environmental and physiological contexts is critical, as microbial activity and metabolite production dynamically adapt to external stimuli [28]. Comprehensive microbiome research requires integrative approaches that combine physiological, metabolic, and pathological data to unravel complex microbial interactions. Additionally, standardized platforms for multi-omics data acquisition and integration are necessary to decode the intricate relationships between gut microbiome ecosystems and host physiology [29].

To address these challenges, three critical research platforms must be developed: (1) a culture-based microbiome ecological analysis platform to study microbial interactions under controlled conditions, (2) an integrative multi-omics platform to analyze and correlate genomic, transcriptomic, proteomic, and metabolomic data, and (3) a microbiome interaction research platform to investigate host-microbiome crosstalk in health and disease.

High-quality, standardized approaches to microbiome data collection and analysis are critical for translating microbiome research into clinical applications. This review explores the evolution of multi-omics technologies and their pivotal role in advancing microbiome research. Furthermore, it discusses future perspectives on how multi-omics can drive precision medicine and personalized nutrition, shaping the next generation of microbiome-based diagnostics and therapeutics.

## Evolution of Microbiome Research

### Overview of Metagenomics as a Pivotal Step

The emergence of DNA sequence-based metagenomics marked a transformative shift in microbiome research, enabling the direct sequencing of genetic material from entire microbial communities [30]. Unlike culture-dependent methods, metagenomics allows for the comprehensive extraction and bioinformatics-driven analysis of microbial DNA, thereby overcoming limitations associated with studying unculturable microbial species [31]. Early culture-based studies provided foundational insights into microbiome structure and function, revealing age-dependent shifts in gut microbiota composition linked to disease phenotypes. For instance, reductions in *Bifidobacteria* and *Bacteroides* were observed in elderly individuals with *Clostridium difficile* infections, highlighting the relationship between microbial composition and host health [32]. However, the inability to culture a significant proportion of microbial species and the restricted scope of early experimental studies necessitated the adoption of 16S rRNA sequencing, which enabled large-scale microbial community profiling. This technique facilitated direct comparisons between mucosal and fecal samples, revealing the intricate microbial ecosystems within the human gut [33].

The integration of metagenomics with next-generation sequencing (NGS) significantly expanded the ability to capture the diversity of previously unculturable gut microbes [34]. A metagenomic study on obese and lean twins, for example, identified a shared “core” gut microbiome at the gene level, demonstrating functional overlaps despite taxonomic differences [35]. This study further established correlations between microbial composition, host genetic factors, and environmental influences on metabolism, illustrating the microbiome’s role in metabolic health. Obesity was found to be associated with phylum-level shifts, reduced bacterial diversity, and enrichment of specific microbial genes related to energy metabolism. The advent of high-throughput (HT) shotgun metagenomics marked a breakthrough, facilitating in-depth microbial community analyses at an unprecedented resolution [34, 36].

**Fig. 1:**
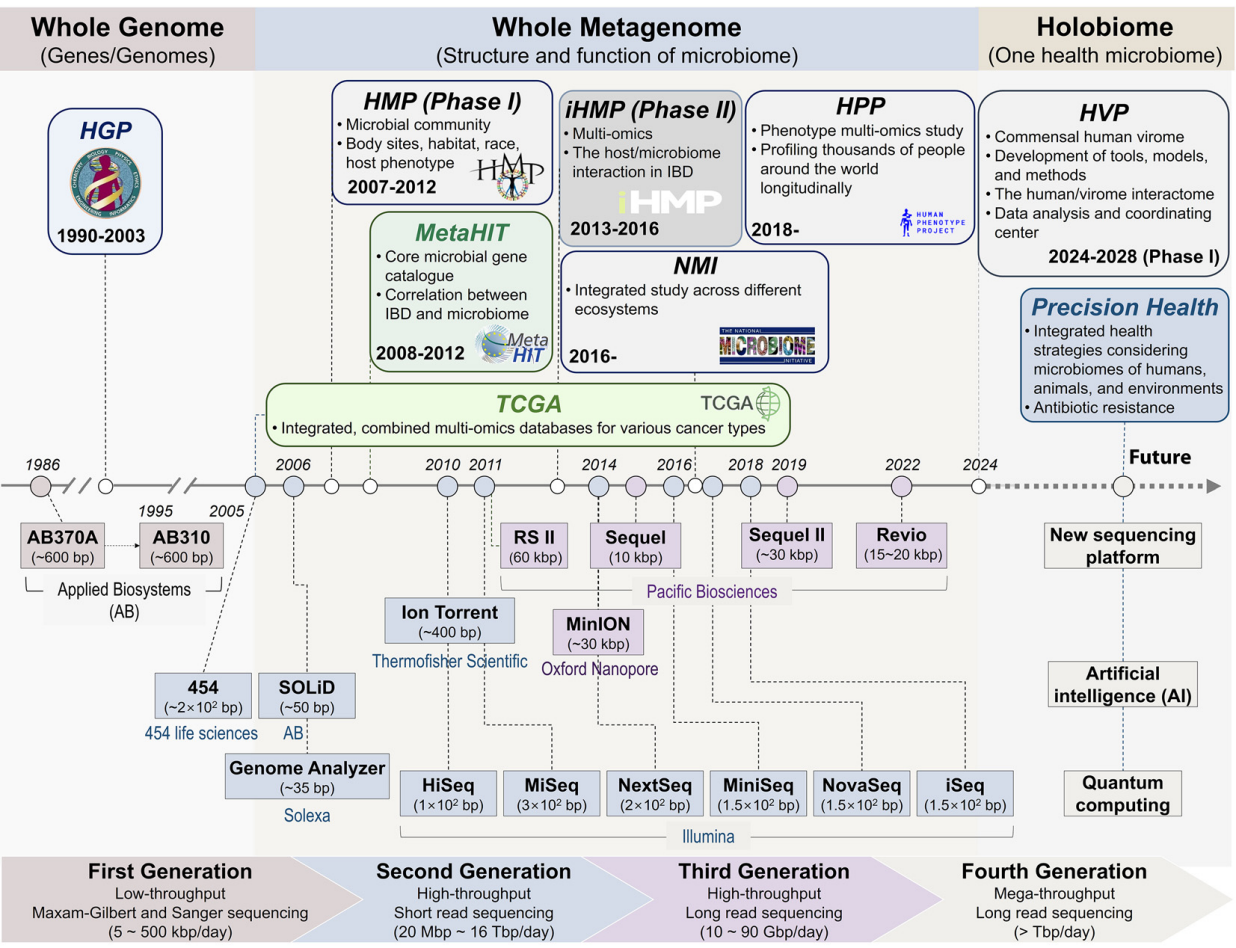
Timeline of major advancements and milestone studies in metagenomics. This timeline depicts significant milestones in human metagenomics research, beginning with the advent of Sanger sequencing, which laid the foundation for microbial genomic analysis. The progression to shotgun sequencing enabled deeper insights into microbial diversity and functions, leading to landmark projects such as the Human Microbiome Project (HMP) Phase I and the Metagenomics of the Human Intestinal Tract (MetaHIT) project. The development of long-read sequencing technologies, as demonstrated by HMP Phase II (also referred to as the integrative HMP or iHMP), facilitated the integration of multi-omics data with clinical metadata. Looking forward, advancements in artificial intelligence (AI) and quantum computing are anticipated to further precision health research and revolutionize metagenomics.

In biomedical research, metagenomics has played a crucial role in identifying key microbial species associated with various diseases [37]. Landmark projects such as the HMP and MetaHIT have significantly advanced microbiome studies by characterizing core microbial species and genes within the human gut microbiome (Fig. 1). These studies demonstrated that while certain microbial genes are highly conserved across individuals to maintain gut homeostasis, microbial composition exhibits considerable variation across populations with differing genetic backgrounds, environmental exposures, and dietary habits [19, 22]. Additionally, within a single individual, microbial communities display niche-specific specialization based on anatomical location, further highlighting the functional and ecological complexity of the microbiomes [19].

Metagenomic studies have also identified individualized patterns of microbial genetic variation, including single nucleotide polymorphisms (SNPs), short insertions and deletions (indels), and structural variants in fecal samples. These findings suggest that microbial profiles remain highly individualized and stable over time, providing a foundation for utilizing the microbiome as a diagnostic marker [37]. However, this stability presents a challenge in efforts to modify microbial profiles through therapeutic interventions.

Another major milestone in metagenomics was the identification of disease-associated microbial signatures, particularly in conditions such as IBD, obesity, and diabetes [22, 25, 38]. For instance, Chatelier *et al*. demonstrated that individuals with obesity-related phenotypes, including insulin resistance, exhibited reduced gut bacterial richness, indicating a potential microbial marker for metabolic disorders [38]. Additionally, studies on type 2 diabetes (T2D) suggest that rather than the presence or absence of a single microbial species serving as a biomarker, “functional dysbiosis”, characterized by a decline in butyrate-producing bacteria, plays a crucial role in disease susceptibility [39]. These findings underscore the value of functional metagenomics in identifying microbial markers for disease diagnosis, risk assessment, and treatment.

Collectively, these foundational discoveries have driven a paradigm shift in microbiome research, transitioning from purely taxonomic descriptions to functional microbiome characterization and host-microbe interactions. This shift has been instrumental in developing microbiome-based diagnostics, advancing precision medicine, and facilitating therapeutic interventions, ultimately deepening our understanding of the gut microbiome’s impact on human health and disease.

### Limitations and Challenges

Despite its transformative impact, metagenomics has inherent limitations that constrain its ability to fully characterize microbial ecosystems. A major challenge is its incomplete capture of the entire microbial ecosystem, which consists not only of bacterial communities but also fungi (mycobiome) [30], viruses (virome) [40], and archaea (archaeome) [41]. Historically, metagenomic studies have focused primarily on bacterial diversity, leading to incomplete representations of the gut microbiome [30, 42]. This limitation is further exacerbated by gaps in reference databases, inconsistent taxonomic classification, and contamination with non-bacterial sequences, all of which reduce the reliability and accuracy of microbial annotations [43].

**Technical biases and methodological challenges.** Metagenomic analyses are subjected to technical biases that can significantly affect results. One of the most widely used methods, 16S rRNA sequencing, relies on PCR amplification, which introduces variability due to differences in target region selection (V1 to V9) [44], primer specificity [45], and PCR-induced artifacts [46]. Additionally, microbial classification based on operational taxonomic units (OTUs) or amplicon sequence variants (ASVs) can yield inconsistent ecological interpretations. Certain bacterial groups, such as Bacilli and Bacteroidia, exhibit poor correlation between these classification methods [47]. Such methodological inconsistencies have contributed to conflicting findings, exemplified by the ongoing debate over the association between the Bacillota (formerly Firmicutes)/Bacteroidota (formerly Bacteroidetes) ratio and obesity [42, 48].

**Challenges in data interpretation.** The vast complexity and volume of metagenomic data present significant analytical challenges [49]. Metrics such as alpha and beta diversity are commonly used to correlate microbial profiles with host phenotypes. However, these measures often oversimplify intricate microbial interactions and require additional functional context for accurate interpretation [50]. Broad taxonomic classifications, such as Bacillota and Bacteroidota, may obscure species-level variations [22], limiting our ability to infer microbial functions and their association with diseases. Consequently, functional pathway analyses and species-specific metabolic profiling have become essential in deciphering microbial contributions to health and disease [51].

**Reproducibility and standardization issues.** A critical challenge in metagenomics is the reproducibility of results across studies. Differences in sample collection protocols, DNA extraction methods, sequencing platforms, and bioinformatics pipelines contribute to substantial variability hindering cross-study comparability [52, 53]. Addressing these inconsistencies requires standardized protocols and reproducible methodologies [34, 53]. Additionally, variations in sequencing depth affect the detection of low-abundance functional genes. For example, identifying antimicrobial resistance genes necessitates a sequencing depth exceeding 80 million reads, underscoring the influence of coverage on data resolution [54].

**Functional redundancy and microbial resilience.** A fundamental challenge in microbiome research is functional redundancy, where multiple microbial species perform similar metabolic functions. This redundancy provides ecological stability, buffering the microbiome against compositional changes and external perturbations [55, 56]. For example, the production of short-chain fatty acids (SCFA) remains stable across diverse carbohydrate substrates, even when microbial composition fluctuates [57]. Consequently, taxonomic shifts do not always result in functional alterations, complicating efforts to infer microbial contributions based solely on compositional data [33].

**Limitations in functional insights and causality.** While metagenomics provides a detailed catalog of microbial genetic potential, it does not assess real-time gene expression or metabolic activity. This limitation makes it difficult to determine which genes are actively transcribed and how microbial communities dynamically interact with their host [58]. Given that microbial metabolism is highly adaptable to environmental changes [28], functional characterization through transcriptomics, proteomics, and metabolomics is essential for capturing dynamic biological processes.

Establishing causal relationships between microbiome composition and disease remains a major challenge. Although many studies have linked microbial imbalances to conditions such as IBD, diabetes, and neurological disorders, distinguishing correlation from causation is difficult [59, 60]. The microbiome is highly individualized and influenced by factors such as diet, environmental exposures, genetics, and lifestyle, making it challenging to identify universal microbial markers for disease prediction [61, 62]. Moreover, microbiome composition can shift rapidly in response to dietary changes, often exceeding inter-individual differences in gene expression, further complicating causal inference [63]. Such short-term variability complicates causal inference, despite the long-term stability of individual microbiomes. Large-scale longitudinal studies with controlled sampling methodologies are necessary to disentangle host-microbiome interactions and validate microbial contributions to disease.

While metagenomics has revolutionized microbiome research, it has intrinsic limitations that hinder a complete understanding of microbial ecosystems. Methodological biases, challenges in data interpretation, and issues related to reproducibility and functional characterization restrict its ability to fully capture microbial complexity. However, integrating metagenomics with transcriptomics, proteomics, and metabolomics through multi-omics approaches is addressing these challenges, providing a more comprehensive framework for studying microbial functions, host interactions, and disease mechanisms. Continued advancements in multi-omics methodologies, coupled with improvements in computational tools and standardized workflows, will further enhance our ability to decode the intricate relationships between the microbiome and human health.

### Recent Advances in Metagenomics

**Fig. 2:**
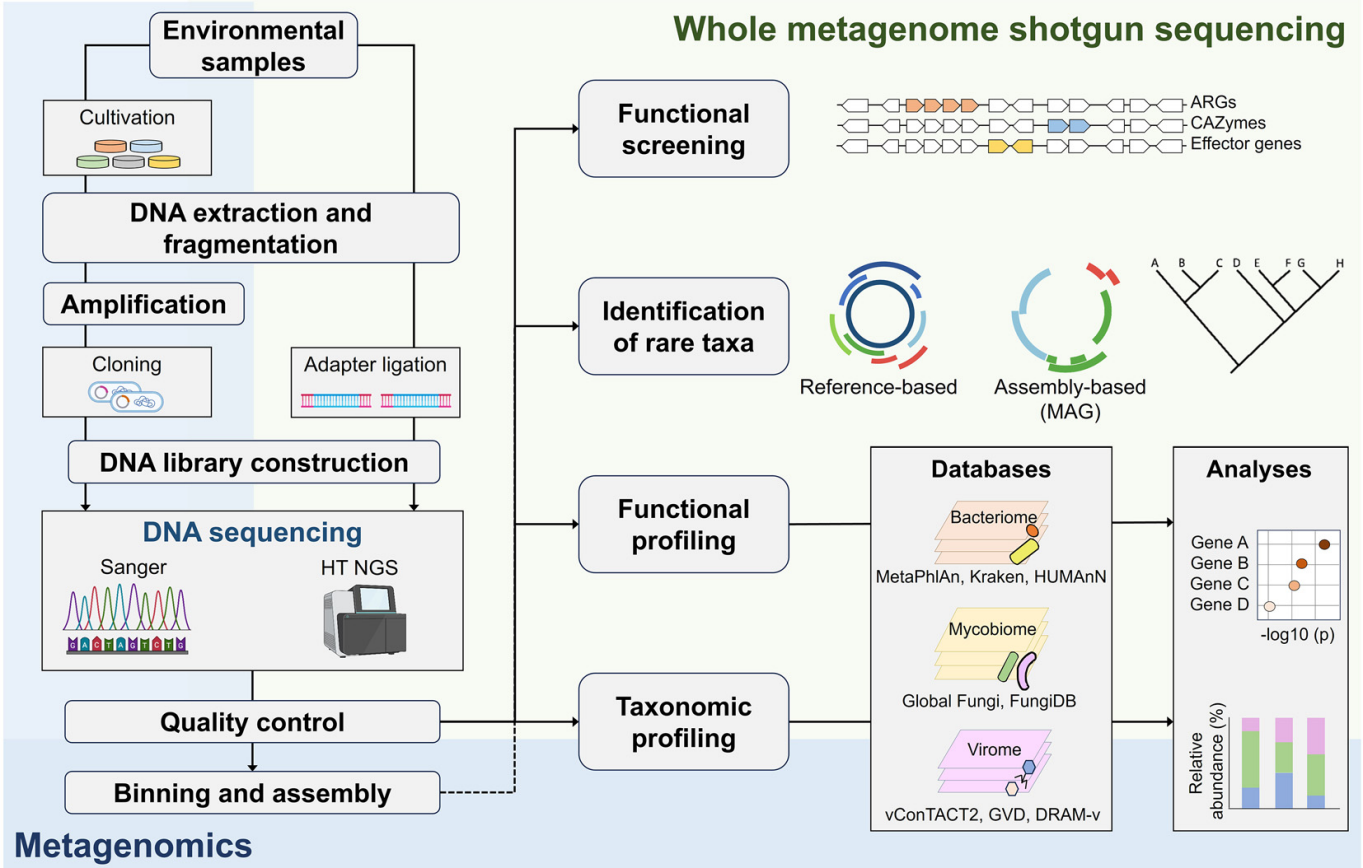
Advancements in metagenomics technologies and methodologies. This figure illustrates the evolution of metagenomic sequencing techniques, starting from traditional methods (colored in blue) to cutting-edge whole metagenome shotgun sequencing (in green). It highlights improvements in sequencing depth, resolution, and data interpretation, which have significantly enhanced the ability to identify taxonomic and functional diversity within microbial communities. Databases and bioinformatics tools, such as the Global Fungi Database (FungiDB) and MetaPhlan, now support these advances, enabling comprehensive microbial profiling.

Advancements in NGS technologies have significantly expanded the scope and efficiency of metagenomics, surpassing traditional culture-based methods (Fig. 2). These innovations enable multi-domain microbial analysis through advanced bioinformatics tools, long-read sequencing, single-cell omics, and enhanced culture-based techniques, facilitating deeper insights into microbial diversity, function, and host interactions. The integration of cutting-edge computational tools and experimental methodologies has enhanced microbiome research, enabling the discovery of novel microbes, metabolic pathways, and therapeutic targets.

**Multi-domain microbial analysis and advanced bioinformatics tools.** Recent metagenomic research has expanded beyond bacterial communities to encompass archaea, fungi, and viruses, fostering a more holistic understanding of the microbiome. Specialized databases such as IMG/VR for viruses [64] and fungiDB for fungal communities [65] facilitate comprehensive taxonomic and functional analyses. Advanced bioinformatics tools further enhance the ability to predict microbial functions and uncover disease-associated elements.

For taxonomic classification, 16S rRNA reference databases, such as SILVA, Greengenes2 (GG2), and the Ribosomal Database Project (RDP) improve the accuracy of amplicon sequencing annotations [66-68]. Functional prediction tools, including PICRUSt/PICRUSt2, Tax4Fun, and FARPROTAX, provide expanded insights into microbial functions by leveraging enhanced genome reference databases [69-72]. Metagenomic shotgun sequencing tools such as MetaPhlAn 3, Kraken2, and HUMAnN 3 allow for high-resolution taxonomic and functional profiling of microbial communities [73, 74]. Additionally, statistical tools such as LEfSe [75], ANCOM-BC [76], and ALDEx2 [77] provide robust microbial abundance analyses.

New metagenomic assembly methods, such as OPERA-MS, have improved the resolution of complex microbial resistomes, biosynthetic gene clusters, and functional gene characterizations [78-80]. To further explore microbial “dark matter”, metagenome-assembled genomes (MAG) utilize advanced computational models, including the overlap-layout-consensus model and De Bruijn graph algorithms, allowing the discovery of novel microbial genomes, as demonstrated in the Genome Taxonomy Database (GTDB) [81, 82]. Despite these advancements, software limitations remain a significant challenge. A recent critical assessment of metagenome interpretation evaluated the performance of various metagenomic analysis softwares (*e.g.*, metaSPAdes, CONCOCT, and MEGAN), revealing that while assembly tools have improved overall performance, they still struggle with resolving closely related strains. Hybrid assembly approaches demonstrated advantages in specific regions, such as the 16S rRNA gene, but did not lead to substantial improvements in overall assembly quality [83]. Additionally, taxonomic binning and profiling showed high accuracy at higher taxonomic levels but exhibited lower resolution at the species level, highlighting the need for further methodological refinements.

**Long-read sequencing and functional metagenomics.** Long-read sequencing technologies, including Oxford Nanopore and Single Molecule, Real-Time (SMRT) sequencing (Pacific Biosciences), have revolutionized metagenomics by providing longer and more accurate reads (Fig. 1). These methods improve genome assembly, particularly in complex gut microbiome samples [84]. For example, extracting high-molecular-weight DNA from stool samples and applying long-read sequencing techniques has yielded highly contiguous microbial genomes [85]. SMRT sequencing enables full-length 16S rRNA profiling [86] and precise circular genome reconstruction [87], even at low sequencing depths [88, 89]. These advancements have been complemented by the rapid development of computational pipelines for genome assembly, taxonomic characterization, and variant detection [90]. Hybrid sequencing approaches that integrate long- and short-read sequencing, such as those used in the Inner Mongolian Gut Genome catalog, provide detailed insights into gut microbiota composition and metabolic capacities [91]. Additionally, long-read sequencing has facilitated the discovery of full-length biosynthetic gene clusters, which are essential for accessing the genetic reservoir of uncultured microbes [92].

**Functional metagenomics for novel gene and pathway discovery.** Functional metagenomics has significantly advanced the discovery of novel genes and metabolic pathways, particularly through HT screening. For instance, this approach has identified genes involved in host-microbe interactions, such as those regulating NF-κB activation, which play a critical role in immune responses [93]. Additionally, innovative screening methodologies, such as Focused Identification of Next-generation Sequencing-based Definitive Enzyme Research (FINDER), have accelerated the discovery of enzyme-specific gene sequences, even in the absence of direct database matches [94]. Within the human gut microbiome, functional metagenomics has uncovered carbohydrate-active enzymes (CAZymes) essential for dietary fiber degradation, underscoring the metabolic versatility of gut microbiota [95, 96].

**Single-cell omics and advanced culture-based methods.** Single-cell omics provides unprecedented resolution for studying microbial diversity and host-microbe interactions. Techniques such as single-cell combinatorial indexing RNA sequencing (SiC-seq) employ droplet microfluidics to isolate, fragment, and barcode individual genomes, allowing unbiased estimations of species abundance [97]. Standardized frameworks, including Minimum Information about a Single Amplified Genome (MISAG) and Minimum Information about a Metagenome-Assembled Genome (MIMAG), have enhanced comparative genomic analyses of individual microbial cells [98].

At the single-cell-transcriptomic level, SiC-seq enables HT functional profiling of thousands of microbial and host cells, providing deeper insights into microbiome-host interactions [99, 100]. Similarly, Cell Expression by Linear Amplification and Sequencing (CEL-seq) has improved the quantification of microbial gene expression, advancing the functional characterization of gut microbiota [101]. A study by Gury-BenAri *et al*. demonstrated the power of single-cell RNA sequencing by revealing distinct immune cell subsets shaped by specific microbial colonization patterns, offering critical insights into immune-microbiome interactions [102].

**Advancing culture-based methods for uncultivable microbes.** Although traditional culture-based approaches often fail to capture the full diversity of gut microbes, advanced culture techniques, when combined with single-cell omics, provide critical insights into viable microorganisms. Single-cell sorting techniques, integrated with genomic tools, now allow researchers to isolate and characterize individual microbial cells [103], overcoming biases associated with relic DNA contamination in metagenomic studies [104].

Emerging techniques such as dilution-to-extinction [81, 105], single-cell sorting [103], and microfluidic HT culturomics [106] have successfully identified novel microbial taxa previously uncultured in laboratory conditions. For instance, single-cell dispensing technology has enabled the isolation and culture of novel microbial species, with up to 31% of recovered isolates classified as potentially new species [103]. Unlike conventional approaches, these methods allow for the identification of slow-growing and low-abundance microbes, which are often overlooked in traditional microbiome studies.

By integrating genomic sequencing with culturomics, researchers can refine taxonomic and functional characterizations, distinguishing healthy microbiomes from dysbiotic ones while accounting for complex microbial interactions [107]. These advancements provide a more complete picture of microbial ecology, host-microbiome interactions, and potential therapeutic applications.

## Multi-Omics: A Holistic Approach

### Multi-Omics Technologies and Their Impact on Microbiome Research

**Fig. 3:**
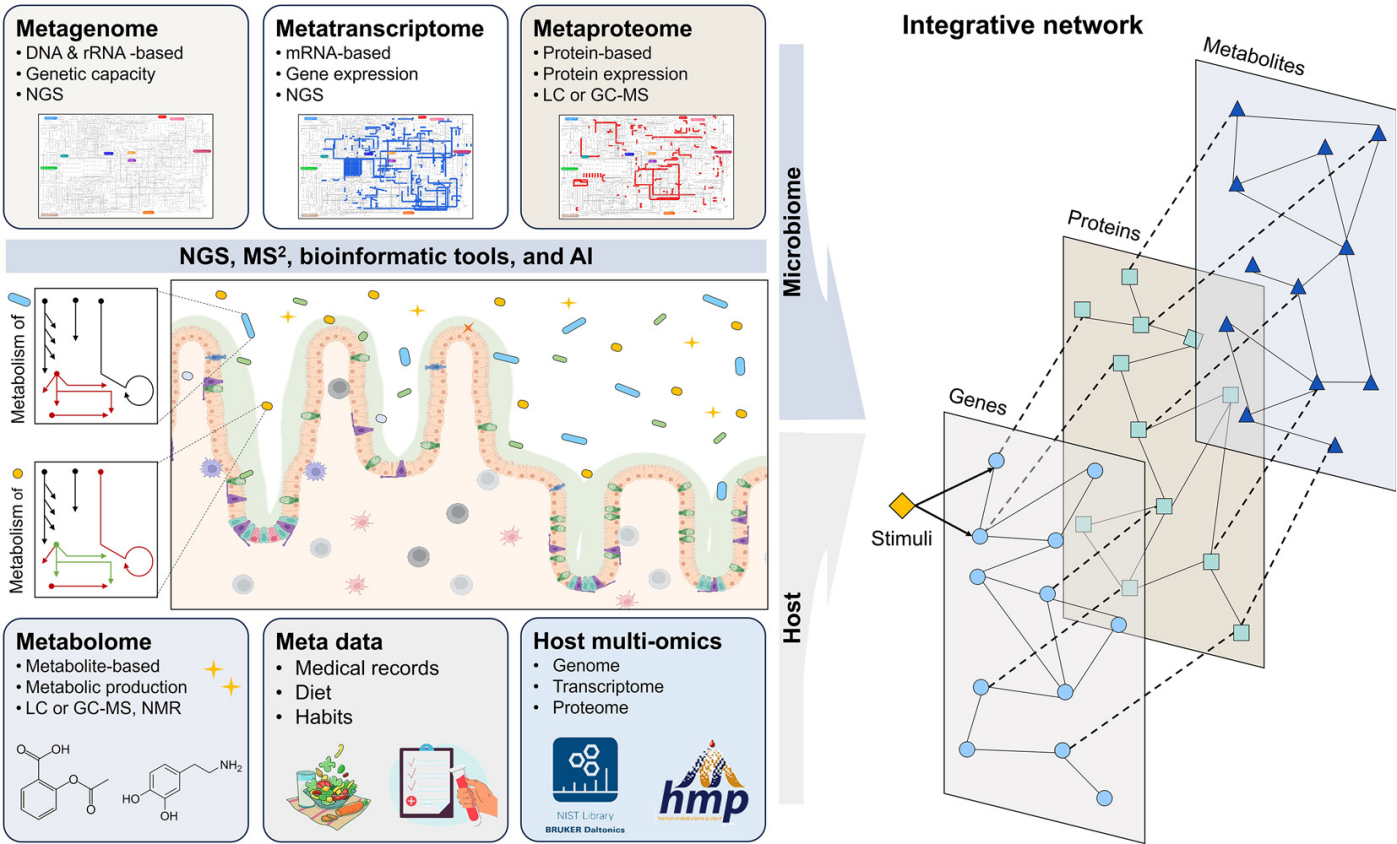
Integration of multi-omics data for understanding host-microbiome interactions. This infographic presents the integration of multi-omics approaches—combining data from metagenomics (DNA and rRNA-based genetic analyses), metatranscriptomics (mRNA-based gene expression), metaproteomics (protein expression and interactions), and metabolomics (metabolic byproducts)—to explore host-microbiome interactions. Advanced technologies such as nextgeneration sequencing (NGS), mass spectrometry (MS), and AI-based bioinformatics are used to process these datasets. Integrated with clinical metadata, this network elucidates how microbial functions and host responses interact in health and disease.

Multi-omics technologies have transformed gut microbiome research by bridging the gap between genetic potential and functional outcomes (Fig. 3). While metagenomics provides valuable insights into microbial genomes and functional capacities, it often fails to capture the dynamic interactions and biological activities within microbial ecosystems [108]. Multi-omics addresses these limitations by integrating transcriptomics, proteomics, and metabolomics, offering a comprehensive and dynamic perspective on biological processes and host-microbiome interactions [109].

**Transcriptomics: Revealing active gene expression in microbial communities.** RNA sequencing (RNA-seq)-based transcriptomics links genetic potential to functional phenotypes by analyzing gene expression patterns in microbial communities [110]. Metatranscriptomics, in particular, identifies actively expressed and regulated genes, shedding light on community-wide biological processes under specific environmental conditions. Notable examples include microbiome-specific phosphonate metabolism in deep-sea environments [111], redox adaptation mechanisms [112], and glycan degradation pathways in ruminants [111].

Integrated metagenomics and transcriptomics studies have further demonstrated the microbiota’s impact on xenobiotic metabolism, such as gut microbial inactivation of cardiac drugs [113, 114], and the upregulation of motility-associated genes in TLR5-deficient mice [115]. Additionally, antisense transcripts have been shown to regulate toxic protein synthesis, highlighting the functional complexity uncovered by transcriptomics [116]. The Men’s Lifestyle Validation Study (MLVS) identified core pathways such as glycolysis and nucleotide biosynthesis, which are universally transcribed, while strain-specific pathways reflect individual microbiome variation [117].

Despite its contributions, metatranscriptomics faces challenges. Prokaryotic mRNA, which constitutes only 1-5% of total RNA and lacks poly-A tails, requires specialized enrichment protocols for accurate analysis [118]. Advances in RNA-seq methodologies, such as those developed by Giannoukos *et al*. [119], have improved RNA extraction and enrichment, enabling more robust prokaryotic gene expression analysis. However, issues such as eukaryotic contamination, the short half-life of mRNA, and discrepancies between mRNA levels and protein expression necessitate integrating transcriptomics with proteomics and metabolomics for a more comprehensive functional perspective [110]. Refined multi-omics protocols now differentiate housekeeping pathways from specialized ones, linking transcriptional activity to microbial ecological functions and host health outcomes [120].

**Proteomics: Mapping functional protein expression in microbial communities.** Proteomics expands upon transcriptomics by directly analyzing the protein composition of microbial communities, providing crucial insights into enzyme activities, host-microbe interactions, and disease-related pathways [121-123]. Metaproteomics studies have identified subject-specific and temporally stable protein profiles over a one-year period, revealing a core set of ~1,000 proteins involved in carbohydrate metabolism and host-microbe interactions. These findings underscore the correlation between microbial activity and individual microbiome composition [124]. Targeted proteomics has further identified active enzymes implicated in inflammation and immune modulation pathways, advancing our understanding of disease mechanisms [125, 126].

One major breakthrough in proteomics is activity-based protein profiling (ABPP), which enables the detection of catalytically active enzymes, such as serine hydrolases and esterases, under host-mimicked conditions. ABPP provides valuable insights into microbial proteins’ roles in both pathogenic and beneficial host interactions [127-129]. Additionally, chemoproteomic approaches have identified reactive cysteine and lysine residues in pathogens, highlighting microbial responses to oxidative stress [130] and revealing proteases and hydrolases that are overrepresented in IBD [131]. Despite significant progress, metaproteomics still faces challenges, including incomplete protein reference databases and discrepancies between protein abundance and mRNA expression levels [132]. Nevertheless, proteomics complements transcriptomics by uncovering functional and spatial protein distributions, enhancing our understanding of disease pathways and therapeutic targets.

**Metabolomics: Linking microbial metabolic outputs to host health.** Metabolomics investigates microbial metabolites, providing real-time insights into microbial phenotypes and metabolic activities [133-135]. Unlike genomic or proteomic data, metabolomic profiles reflect actual metabolic outputs, allowing researchers to study microbial interactions with the host and responses to environmental changes. Early nuclear magnetic resonance (NMR)-based metabolomic studies mapped complex symbiotic interactions between the microbiome, host, and diet, demonstrating the dynamic nature of microbial metabolism [136]. Recent advancements in high-resolution mass spectrometry, small-molecule databases, and machine-learning (ML) algorithms have significantly improved metabolite detection and flux analysis, enabling the identification of metabolic shifts in response to environmental stimuli [137-140]. However, metabolomics alone lacks the genomic and proteomic context, making it difficult to link observed metabolic outputs to their genetic and protein-based mechanisms [141]. To address this, integrating metabolomics with transcriptomics and proteomics is essential for comprehensive functional insights into microbial ecosystems.

**An integrated view of the microbiome through multi-omics.** While each single-omics approach has advanced microbiome research, integrating multi-omics provides a holistic view of microbial functions and host interactions. By combining genomics [142], transcriptomics [108], proteomics [143], metabolomics [143], culturomics [144], and epigenomics [145], multi-omics comprehensively captures microbial community dynamics (Fig. 3). For example, integrating metagenomics with metatranscriptomics correlates genetic potential with actual gene expression, revealing which genes are active under specific conditions [135]. Metaproteomics and metabolomics further link gene expression to protein production and metabolic outputs, offering deeper insights into microbial contributions to host health [146].

The successful integration of multi-omics data relies on advanced bioinformatics platforms that facilitate data processing, interpretation, and visualization. MetaTrans enables comprehensive RNA-seq functional analysis, allowing researchers to examine microbial transcriptomic activity across diverse conditions [147]. Similarly, anvi’o integrates genomic and transcriptomic datasets, providing a multi-layered visualization of microbial community functions [148]. In proteomics, MetaLab automate peptide and taxa profiling from mass spectrometry data, streamlining large-scale proteomic and metaproteomic analyses [149]. Meanwhile, metabolomics tools such as MIMOSA2 [150] and machine learning-based mmvec facilitate microbiome-metabolome association analyses, advancing disease research and therapeutic development [151]. These computational tools have significantly improved the ability to derive biologically meaningful insights from complex multi-omics datasets, bridging the gap between raw data and clinically relevant findings.

Multi-omics approaches have revealed key microbial functions that influence host metabolism, immune responses, and disease susceptibility. For instance, metagenomic and metatranscriptomic integration has highlighted the role of SCFA-producing bacteria as key modulators of immune regulation, emphasizing their contribution to gut homeostasis [152]. Additionally, metabolomics studies have demonstrated microbial contributions to obesity by linking bacterial community shifts to alterations in host metabolic pathways [153]. Studies on diet-induced obesity models have further shown that gut and liver microbiome alterations are accompanied by significant metabolic shifts, reinforcing the role of diet in shaping host-microbiome interactions [154]. By integrating functional genomics, proteomics, and metabolomics, researchers can identify biomarkers, therapeutic targets, and potential microbiome-based interventions for metabolic and immune disorders.

Multi-omics technologies have propelled microbiome research beyond static taxonomic profiling, offering dynamic insights into microbial functional roles, metabolic activities, and host interactions. By integrating genomics, transcriptomics, proteomics, and metabolomics, multi-omics elucidates complex biological processes, enabling breakthroughs in precision medicine and microbiome-based therapies. These integrative approaches are essential for translating microbiome research into clinically actionable insights, ultimately shaping disease diagnostics, therapeutic interventions, and personalized healthcare strategies.

### The Role of Multi-Omics in Linking Microbiome and Therapeutics

Advancements in multi-omics technologies have revolutionized our understanding of host-microbiome interactions, providing critical insights into how microbial imbalances influence health, disease progression, and drug efficacy [155-159]. By integrating genomics, transcriptomics, proteomics, and metabolomics, multi-omics enables a comprehensive analysis of microbial and host dynamics, identifying key pathways that drive disease development and therapeutic responses [160, 161]. This approach allows for the identification of active genes, proteins, and metabolites associated with disease states, allowing for a deeper understanding of microbial contributions to pathology. For example, a multi-omics study integrating metagenomic, metatranscriptomic, and metaproteomic data in four families with type 1 diabetes (T1D) demonstrated that reductions in exocrine pancreatic proteins correlated with microbial activity and specific metabolic traits, providing mechanistic insights into disease progression [162]. Similarly, in IBD, multi-omics analyses identified *Bacteroides*-specific serine and metalloprotease activities as key contributors to disease progression, revealing a direct link between microbial metabolism and immune dysregulation [163]. Furthermore, integrating multi-omics has enabled the identification of disease-specific microbial markers, supporting precision interventions. For instance, metagenomics combined with metabolomics has uncovered co-variations between microbial taxa and host metabolites, providing actionable insights for therapeutic development.

Multi-omics approaches have also been instrumental in therapeutic discovery and biomarker identification [154, 161]. The Human Phenotype Project (HPP), a large-scale longitudinal study that collected extensive clinical and biomolecular data over five years, enabled diverse microbiome research by integrating multi-omics datasets to investigate the relationships between microbiome composition, clinical phenotypes, and molecular features [164, 165]. Alongside this initiative, multi-omics studies have examined microbiome-associated changes in various conditions, including preterm birth, IBD, and T2D [25, 166, 167]. Notably, multi-omics research on irritable bowel syndrome (IBS) has identified symptom-associated microbial compositions and functional phenotypes, underscoring the potential of this approach to uncover disease-specific microbial markers in heterogeneous conditions [168, 169]. In ulcerative colitis (UC), multi-omics analyses uncovered microbial enzymes and metabolites linked to disease severity, including elevated *Phocaeicola vulgatus* (formerly *Bacteroides vulgatus*) protease expression and increased dipeptides and oligopeptides, which contribute to mucosal inflammation and barrier dysfunction. Moreover, in oncology, multi-omics strategies have uncovered novel connections between gut microbiota alterations and changes in host metabolites and gene expression patterns, specifically in region-specific colorectal cancer [62, 170]. By constructing networks that link microbial taxa, metabolites, and tumor-associated genes, these studies provide insights into the potential role of the microbiome in tumorigenesis and its implications for personalized cancer therapy. Collectively, these findings identify key microbial and host factors driving disease progression, while also revealing unique microbial signatures that could serve as diagnostic and therapeutic targets [21, 171].

Beyond disease diagnostics, multi-omics has illuminated the bidirectional relationship between drugs and the microbiome, showing how microbial metabolism affects therapeutic efficacy and toxicity. HT screening studies have shown that nearly 24% of non-antibiotic drugs inhibit gut bacterial species, emphasizing the importance of considering drug-microbiome interactions in therapeutic design [13]. Research on metformin, for example, has highlighted its dual role in modulating gut microbiota composition while simultaneously improving glucose metabolism, demonstrating the potential to repurpose existing drugs based on microbiome-based therapeutic strategies [172]. In addition to drug-microbiome interactions, multi-omics has significantly advanced microbiome-based therapies, such as fecal microbiota transplantation (FMT) and next-generation probiotics, which aim to restore microbial balance in dysbiosis-related disorders. Given that individual variations in gut microbiota composition significantly influence responses to dietary interventions, multi-omics research is paving the way for the development of personalized nutrition plans tailored to an individual’s unique microbial profile [17, 173-175]. These insights offer substantial potential for optimizing therapeutic strategies targeting the microbiome.

In summary, multi-omics technologies are reshaping microbiome research by elucidating microbial activities linked to disease mechanisms and therapeutic responses. These integrative approaches hold great promise for advancing precision medicine by fostering innovative strategies for the diagnosis, treatment, and prevention of microbiome-associated diseases. By linking host-microbe interactions to functional outcomes, multi-omics presents a transformative framework for developing targeted interventions that address both microbial imbalances and host metabolic dysfunctions, ultimately leading to improved patient care.

### Emerging Trends and Future Directions in Multi-Omics

Emerging multi-omics technologies are transforming microbiome research by providing deeper insights into microbial ecosystems, their functional roles, and interactions with the host. One notable advancement is spatial multi-omics, which facilitates the study of microbial spatial organization and interactions within their native environments [176, 177]. This approach is particularly significant in gut microbiome research, where microbial distribution patterns influence functional roles and host interactions [178]. Integrating spatial multi-omics with single-cell technologies offers unprecedented resolution, enabling the identification of specific cell types and subpopulations responsible for critical biological functions [179, 180]. For example, spatial characterization of the mouse gut microbiome has revealed heterogeneous distributions of individual taxa, illustrating diverse co-associations and the impact of dietary shifts on local phylogenetic diversity and microbial interactions. These findings highlight the ecological and functional relevance of spatial analyses in deciphering microbiome complexity.

Another major development is the expansion of real-time, longitudinal, large-scale cohort studies, which facilitate continuous monitoring of microbiome dynamics over time. These studies provide crucial insights into how microbial communities respond to environmental and clinical factors, including dietary interventions and pharmaceutical treatments. For instance, a longitudinal multi-omics study investigating antibiotic-induced perturbations elucidated post-treatment microbiome recovery dynamics, identifying key factors influencing microbial resilience and susceptibility [181]. Similarly, the TEDDY study, which analyzed over 20,000 sequencing datasets from 903 children, identified developmental phases of the early gut microbiome [182] and their associations with breast milk composition and the onset of T1D onset [183]. These findings underscore the importance of large-scale, longitudinal studies in mitigating individual variability and advancing our understanding of microbiome stability and its implications for health and disease [184].

**Fig. 4:**
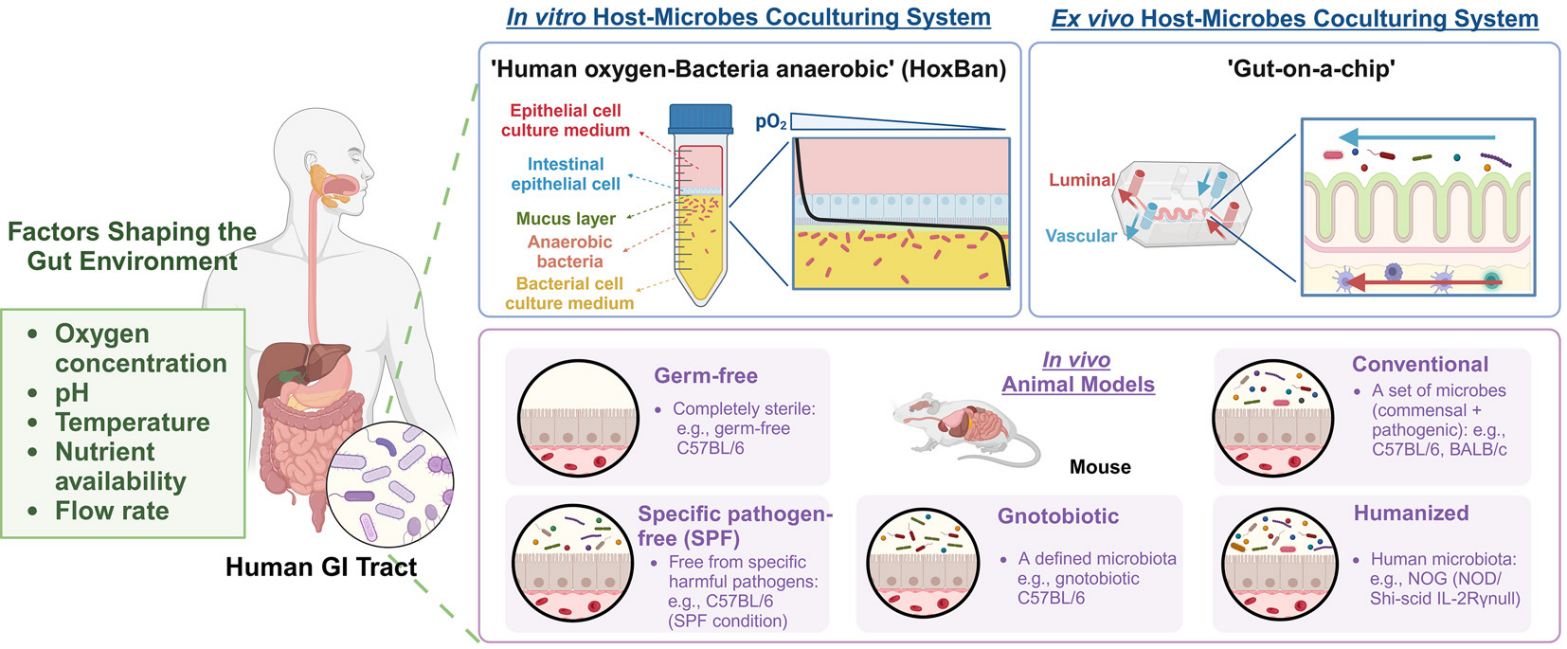
Experimental platforms for mimicking host-microbiome interactions. This figure illustrates three experimental platforms for studying gut microbiome dynamics: (1) *In vitro* systems, such as the Human Oxygen-Bacteria Anaerobic (HoxBan) system, which enables coculturing of oxygen-requiring human cells and anaerobic gut bacteria to analyze metabolic and transcriptomic exchanges; (2) *Ex vivo* models, such as gut-on-a-chip systems that simulate intestinal conditions using microfluidics; and (3) *In vivo* models, including germ-free, gnotobiotic, and humanized mice, which replicate hostmicrobiome interactions within live organisms. These platforms provide tools for studying the gut environment under controlled and physiologically relevant conditions. This figure was created in BioRender. Han, S. (2025) https://BioRender.com/u46p776.

**Development of experimental systems and platforms.** While multi-omics approaches have provided profound insights into microbiome functions, sequencing and mass spectrometry data alone are insufficient to fully elucidate microbial roles or metabolic activities. To address these limitations, researchers have developed experimental platforms that integrated culture techniques with host-mimicking systems, enabling controlled investigations of microbial and host responses (Fig. 4).

One such platform is the human oxygen-bacteria anaerobic (HoxBan) coculture system, which supports the simultaneous growth of oxygen-dependent human cells and anaerobic gut bacteria. This system facilitates detailed analyses of metabolic exchanges and transcriptomic profiles. For example, co-culturing *Faecalibacterium prausnitzii* with Caco-2 cells stimulated bacterial growth while exhibiting anti-inflammatory and antioxidant effects, providing key insights into microbial-host interactions [185]. Additionally, mouse models, including germ-free, gnotobiotic, and humanized mice, have been instrumental in elucidating the role of microbes in host physiology and disease progression [186]. These models allow researchers to investigate disease mechanisms, such as IBD, using inflammation-inducing agents like dextran sodium sulfate (DSS), which disrupts the colonic epithelial barrier [187], and 2,4,6-trinitrobenzene sulfonic acid (TNBS), which induces acute T cell-mediated intestinal inflammation driven by IL-12 [188]. Furthermore, IL-10-deficient mice serve as widely used models for UC, offering valuable insights into T-cell-mediated immune responses and disease pathogenesis [189].

In addition to *in vivo* models, gut-on-a-chip technology has emerged as a powerful tool for studying gut microbiome interactions in controlled environments [190]. These microfluidic devices replicate key physiological conditions, including oxygen gradients across the intestinal lumen, while maintaining microbial diversity. By providing a stable and adaptable platform, gut-on-a-chip systems allow researchers to explore host-microbiome dynamics in response to dietary, pharmaceutical, and environmental interventions [191]. These innovative platforms collectively expand the capacity to investigate microbiome-driven mechanisms underlying health and disease, offering valuable translational insights for precision medicine.

**Remaining challenges in multi-omics research.** Despite its transformative potential, multi-omics research faces several challenges. Multi-omics approaches are resource-intensive, requiring substantial financial and computational investments compared to single-omics methods. The generation of large, complex datasets necessitates advanced bioinformatics tools for effective integration and interpretation. However, integrating data across multiple omics layers (*e.g.*, genomics, transcriptomics, proteomics, and metabolomics) remains challenging due to discrepancies in data types, scales, and temporal resolution [192]. Additionally, standardized protocols and robust bioinformatics pipelines are essential to ensure reproducibility and comparability across studies.

Addressing these challenges will require continued advancements in computational methodologies, data harmonization strategies, and cost-effective sequencing technologies. Furthermore, interdisciplinary collaborations will be crucial in integrating multi-omics data with clinical metadata, ultimately translating microbiome research into actionable therapeutic and diagnostic solutions. As multi-omics continues to evolve, these innovations will pave the way for a deeper understanding of microbiome-host interactions and their role in health and disease, driving the next generation of precision medicine and microbiome-based therapeutics.

## Integrative Approaches in Microbiome Research

### Importance of Data Integration

While multi-omics technologies provide a comprehensive understanding of microbial functions, integrating clinical, demographic, and environmental data is essential for achieving a holistic perspective on the microbiome’s role in health and disease. Incorporating patient health records, dietary habits, and environmental exposures enhances the identification of microbial signatures and functional pathways associated with clinical conditions, facilitating the development of targeted and personalized interventions [193-196]. For instance, integrative studies have identified microbial biomarkers for diseases such as T2D and IBD, demonstrating how genetic, environmental, and dietary factors influence disease pathogenesis [21, 167]. Environmental factors, including stress and antibiotic use, also play a crucial role in shaping gut microbiome dynamics. Studies that integrate multi-omics data with clinical information, particularly in cases of antibiotic-induced microbiome perturbation, have provided critical insights into microbiome resilience and susceptibility, revealing how patient-specific factors influence recovery and long-term microbiome stability [197, 198].

Key bioinformatics methodologies, such as data normalization, dimensionality reduction, network analysis, and machine learning, are essential for identifying patterns and correlations within multi-omics datasets. These analytical tools facilitate the integration of omics data with clinical and environmental information, allowing researchers to draw meaningful conclusions about the microbiome’s impact on health and disease progression [199]. While databases such as The Cancer Genome Atlas (TCGA) [200] and the Omics Discovery Index [201] provide valuable frameworks for multi-omics data integration, they primarily focus on specific diseases and often exclude microbiome datasets, limiting their applicability to microbiome research (Fig. 1). The future of microbiome research lies in the seamless integration of diverse datasets, supported by advanced bioinformatics platforms for real-time analysis, spatially resolved omics, single-cell technologies, and host-specific non-omics data. These advancements will significantly deepen our understanding of microbiome-host interactions and drive innovations in precision healthcare solutions.

### Challenges in Data Integration

Despite its immense potential, integrative microbiome research faces several challenges, including data heterogeneity, methodological variability, and limited computational infrastructure, which hinder progress. Multi-omics datasets are generated in diverse formats, often lacking universally accepted workflows for integration [202]. Differences in DNA/RNA extraction, protein/metabolite processing, and bioinformatics pipelines frequently lead to inconsistent findings and hinder reproducibility across studies. Additionally, non-technical biases, such as regional and cultural variations in dietary habits, genetic background, and lifestyle, introduce further complexities [203, 204]. Given the strong influence of these factors on gut microbiota composition, discrepancies may emerge when integrating datasets from different populations. Addressing these methodological and biological variations is critical for ensuring data reliability and comparability, particularly in global-scale microbiome research.

To overcome these challenges, the establishment of standardized platforms for data collection, analysis, and interpretation is essential [205]. These platforms should incorporate uniform protocols for sample processing, sequencing, and data analysis, as well as standardized data formats to facilitate cross-study comparisons and data sharing [206]. Centralized databases that integrate multi-omics, clinical, and environmental data will be crucial in enabling large-scale meta-analyses. For example, the Earth Microbiome Project (EMP) demonstrated the importance of consistent protocols in re-characterizing microbial community samples, enabling more robust and reproducible meta-analyses [207]. These initiatives underscore the urgent need for updated reference databases and harmonized frameworks, which are essential for advancing microbiome research and its clinical applications.

By addressing these challenges and leveraging technological advancements, integrative microbiome research has the potential to revolutionize precision medicine, facilitating the development of personalized interventions that account for both host-specific and microbial factors. The continued refinement of computational tools, bioinformatics pipelines, and standardized workflows will be critical in unlocking the full potential of multi-omics-driven microbiome research for human health and disease management.

### Applications of Artificial Intelligence and Machine Learning in Data Integration

The rapid advancement of HT sequencing and multi-omics technologies has led to the generation of vast, complex datasets that require sophisticated computational tools for integration and interpretation. Artificial intelligence (AI) and ML have become indispensable for extracting meaningful insights from microbiome data [208], offering predictive capabilities that reveal microbial interactions and their relationships to health outcomes [209]. ML models are particularly valuable in integrating multi-omics datasets, identifying key microbial signatures, and predicting disease phenotypes [210, 211].

In microbiome research, ML-based models have successfully predicted disease status and identified microbial features associated with conditions such as T2D and IBD by analyzing metagenomic and metabolomic data [161, 212]. For instance, ML frameworks analyze microbial features as input, assess model performance using methods like random forest, and interpret results with tools such as Shapley additive explanations (SHAP). Additionally, ML-driven tools for predicting microbial metabolite production from genome sequences have been developed, significantly enhancing our ability to understand microbiome-metabolome interactions and their implications for disease [151, 213, 214]. By employing computational learning networks, such as neural networks, ML models can predict microbiome-metabolome axes, thereby uncovering relationships between gut microbiota, microbe-derived metabolites, and disease pathways.

Beyond traditional ML approaches, deep learning (DL) is being explored as a powerful tool for deciphering complex microbiome data. Unlike standard ML models, DL architectures can recognize intricate nonlinear patterns in large-scale dataset [215]. However, DL models require significantly larger sample sizes for training and are more challenging to interpret, making their clinical applicability more complex than conventional ML approaches. Nonetheless, efforts to refine DL methodologies are ongoing. For example, Shen *et al*. recently developed a DL-based pipeline, MEGMA, which successfully identified microbial biomarkers across five disease datasets [216]. Such AI-driven advancements hold great promise for enhancing diagnostic accuracy, identifying microbial markers with clinical significance, and accelerating microbiome-based therapeutic applications.

Despite its potential, ML and DL approaches face several limitations. Complex models require substantial computational resources, and their outputs often reflect correlations rather than causal relationships, raising concerns about interpretability and reliability [209]. Additionally, data quality issues such as biases, duplications, and missing information, can compromise the accuracy of model predictions. Addressing these challenges will require continuous refinement of ML methodologies, improved data standardization, and the development of robust validation frameworks. As AI-driven tools become more sophisticated, they will play an increasingly pivotal role in advancing microbiome research, precision medicine, and microbiome-targeted therapeutic strategies [211].

## Trends in Microbiome Research toward Precision Medicine

As microbiome research progresses, emerging trends are shaping its future, particularly in precision medicine and personalized nutrition. One of the most transformative developments is the integration of multi-omics technologies, which enables personalized healthcare by analyzing individual microbiome profiles. This approach identifies microbial signatures linked to specific diseases and therapeutic responses, enabling targeted interventions that surpass the limitations of a one-size-fits-all model, significantly improving patient outcomes [217].

The emergence of personalized nutrition has further accelerated the application of multi-omics technologies in probiotics research [218]. Multi-omics approaches have advanced our understanding of host-probiotic interactions, providing mechanistic insights into their role in disease modulation and therapy optimization. For instance, studies on *Bifidobacterium bifidum* have demonstrated its ability to enhance cancer treatment efficacy, underscoring the potential of probiotics in personalized medicine [219]. These findings will inform the development of precision-engineered probiotic formulations tailored to individual gut microbiota compositions, optimizing therapeutic outcomes.

Microbiome engineering has emerged as a promising strategy, leveraging synthetic biology and gene-editing technologies, such as Clustered Regularly Interspaced Short Palindromic Repeats (CRISPR), to precisely modify microbial genomes [220, 221]. These tools enable the design of custom microbial consortia with tailored functional properties, facilitating targeted microbiome restoration in conditions such as dysbiosis and other microbiome-associated diseases [222]. This approach holds immense potential for correcting pathogenic microbial imbalances and enhancing host-microbe interactions to improve health outcomes.

Advancements in systems biology and computational modeling are further expanding the frontiers of microbiome research. Technologies such as BASEHIT, which enables proteome-scale screening of interactions between human-associated bacteria and human exoproteins, have uncovered nearly two million potential interactions relevant to health and disease [223]. Similarly, Transkingdom Network Analysis (TkNA) integrates multi-omics datasets to reconstruct complex host-microbiome interaction networks, providing a more comprehensive systems-level perspective and identifying causal relationships between microbial communities and host physiology [224].

**Fig. 5:**
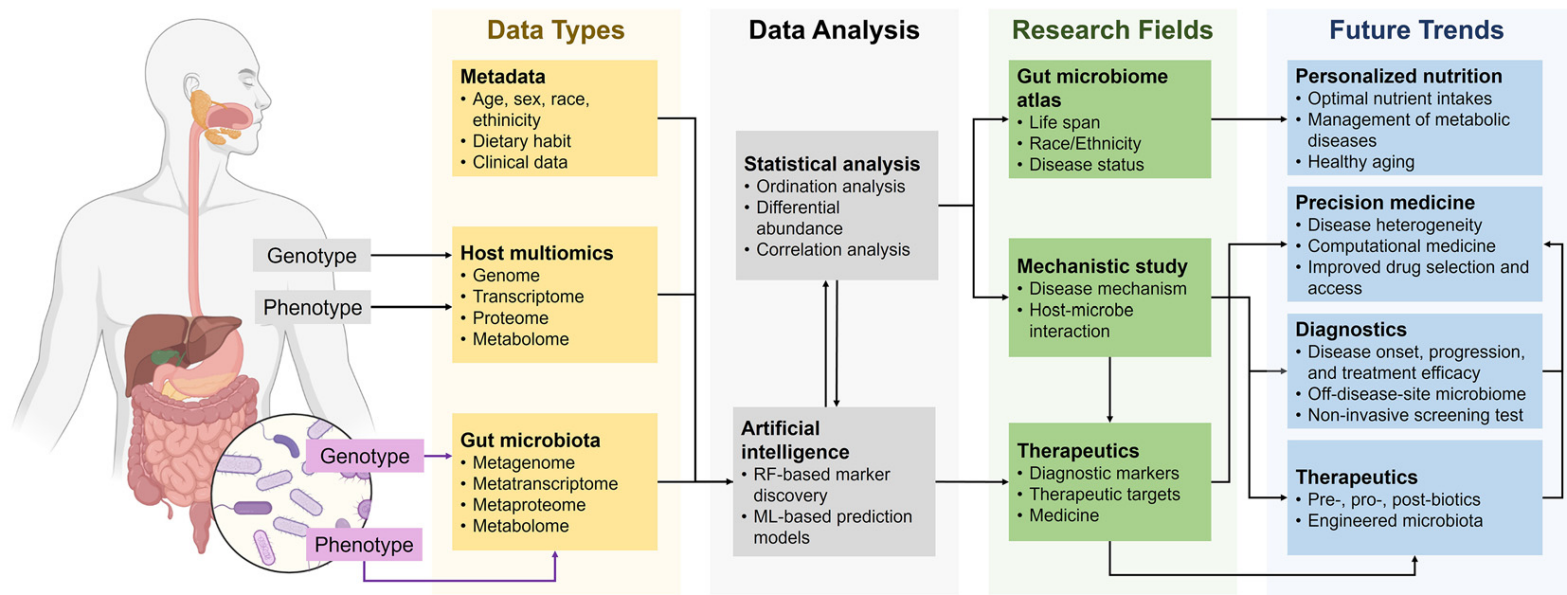
A schematic framework for progress in microbiome research. This framework highlights how microbiome research integrates multi-omics data (yellow) and clinical metadata to uncover insights into host-microbe interactions. Computational analyses (gray) enable diverse research applications, such as mapping disease mechanisms, developing therapeutics, and advancing diagnostic tools (green). These findings feed into emerging trends (blue), including personalized nutrition, precision medicine, and novel diagnostics, guiding the future of microbiome-based healthcare innovations.

Looking ahead, microbiome research will focus on deciphering the intricate crosstalk among microbial and host biological molecules [225]. A holistic systems biology approach will be essential to capturing the dynamic processes of microbial ecosystems and their adaptive responses to varying environmental and host conditions (Fig. 5). By analyzing multi-omics interactions, researchers can elucidate how diverse biological layers collectively influence health and disease, paving the way for highly precise therapeutic interventions. Realizing these objectives will necessitate substantial advancements in data integration, with enhanced computational tools and machine learning algorithms playing a crucial role in linking microbial gene expression, protein synthesis, and metabolic activity with host physiology and disease outcomes. These integrated strategies will not only deepen our understanding of the microbiome but also drive the development of next-generation diagnostics and therapeutics, marking a pivotal step toward truly personalized medicine.

## Conclusion

The transition from metagenomics to multi-omics has fundamentally transformed our understanding of the gut microbiome, providing unprecedented insights into microbial functions and host interactions. While metagenomics established the foundation for studying microbial community structures, multi-omics has extended this knowledge by providing a dynamic, system-level perspective, revealing how microbial communities interact with their environments. This paradigm shift has created new opportunities for precision medicine, allowing for personalized healthcare strategies that leverage individual microbial profiles.

Despite these advancements, several critical challenges remain. Fundamental questions about how a balanced microbiome is maintained through host-microbe co-evolution and how individual variations in microbiota influence health and disease remain unresolved [226]. Addressing these challenges will require continued innovation in sequencing technologies, bioinformatics tools, and data integration methodologies, which are vital for further unraveling the complexities of the microbiome and translating microbiome research into clinical applications.

Looking forward, the potential of multi-omics to revolutionize healthcare is immense. By offering a comprehensive understanding of microbial ecosystems and their intricate interactions with the host, multi-omics presents new opportunities for precise diagnostics, targeted therapies, and disease preventive strategies. The continued integration of diverse datasets, refinement of analytical tools, and responsible application of these technologies in clinical settings will be crucial for advancing microbiome-based precision medicine. As personalized medicine becomes increasingly viable, microbiome-informed treatments tailored to individual microbial and genetic profiles will lead to improved health outcomes, ushering in a new era of effective, sustainable, and personalized healthcare.
